# Minimum-Run Resolution IV Design for Optimized Bio Removal of Fe^2+^ Using *Enteromorpha intestinalis* Aqueous Extract and Its Extract-Coated Silver Nanoparticles

**DOI:** 10.3390/plants14010040

**Published:** 2024-12-26

**Authors:** Doaa G. El-Hosari, Fatma A. Mokhtar, Hussein A. Khalaf, Ahmed R. N. Ibrahim, Rehab M. Mohamed, Mofida E. M. Makhlof

**Affiliations:** 1 Department of Pharmacognosy, Faculty of Pharmacy, Helwan University, Ein Helwan, Cairo 11795, Egypt; 2 Department of Pharmacognosy, Faculty of Pharmacy, El Saleheya El Gadida University, El Saleheya El Gadida 44813, Egypt; 3 Fujairah Research Centre, Sakamkam Road, Sakamkam, Fujairah 00000, United Arab Emirates; 4 Chemistry Department, Faculty of Science, Damanhour University, Damanhour 22511, Egypt; hkhalaf70@sci.dmu.edu.eg; 5 Department of Clinical Pharmacy, College of Pharmacy, King Khalid University, Abha 61421, Saudi Arabia; aribrahim@kku.edu.sa; 6 Biological and Geological Sciences Department, Faculty of Education, Ain Shams University, Al-Maqrizi Street, Roxy, Cairo 11341, Egypt; rehabmostafa@edu.asu.edu.eg; 7 Botany and Microbiology Department, Faculty of Science, Damanhour University, Damanhour 22511, Egypt; mofida_makhlof@sci.dmu.edu.eg

**Keywords:** green synthesis, bioremoval, Fe^2+^, *Enteromorpha intestinalis*, silver nanoparticles

## Abstract

Biosorbents have demonstrated considerable potential for the remediation of metals in aqueous environments. An aqueous extract of *Enteromorpha intestinalis* L. (EiE) and its extract-coated silver nanoparticles have been prepared and employed for the removal of iron. Fourier transform infrared spectroscopy (FTIR), X-ray diffraction (XRD), UV-visible spectroscopy, transmission electron microscopy (TEM), gas chromatography-mass spectroscopy (GC-MS), and zeta potential were employed to characterize the prepared biosorbents. The adsorption properties of the biosorbents were investigated in batch experiments, with a range of factors taken into account, including pH, contact time, initial ion concentrations, biosorbent dosage, and temperature. A minimum-run resolution IV design (MRR-IV) was developed with the objective of optimizing the removal efficiency. The mechanisms of adsorption were investigated using both the Langmuir and Freundlich isotherms. Kinetic studies were conducted using the pseudo-first-order and pseudo-second-order models. A variety of active constituents, including organic acids, lipids, alcohols, and terpenes, were identified through the use of GC-MS, with the findings supported by FTIR spectra. Transmission electron microscopy (TEM) revealed that the nanoparticle size ranged from 5 to 44 nm, while X-ray diffraction (XRD) demonstrated a high degree of crystallinity. A screening study employing the MRR-IV methodology, facilitated by the Design-Experiment, Ver 13., indicates that three factors exert a considerable influence on the biosorption process. The study demonstrated that the biosorption mechanism is pH-dependent, with an optimal pH of 5. The adsorption performance was found to follow Freundlich isothermal models and pseudo-first-order kinetics.

## 1. Introduction

Water contamination due to urbanization and population growth is a pressing environmental concern. The introduction of waste into aquatic systems has resulted in a significant decline in water quality, with metals being prominent contaminants in surface, ground, and marine environments [1,2,3]. Metal ions in water are particularly worrisome due to their toxicity, bioaccumulation through the food chain, and potential to harm aquatic ecosystems and human health [4,5]. While various removal methods such as ion exchange, chemical precipitation, reverse osmosis, and adsorption have been explored, many have limitations, including high costs and inefficiency in low-concentration scenarios [6,7]. Among these, adsorption has emerged as a preferred method due to its cost-effectiveness, simplicity, and adaptability [8].

Iron, one of the most abundant metals, poses significant challenges when present in excessive quantities in water, causing discoloration, staining, and adverse effects on water quality and treatment processes. Excessive iron can also lead to severe health risks such as metabolic syndrome and cardiovascular diseases [9,10,11]. Many techniques, including ion exchange, coagulation/flocculation, flotation, photocatalysis, solvent extraction, electro remediation, biological sludge, adsorption, and membrane technology, have been used to remove metal ions from solutions [12]. Traditional methods for iron removal have varying efficacy, but adsorption using biomass-derived materials has shown promise due to its environmental sustainability and economic viability [13,14,15,16]. Algae, particularly seaweed, have demonstrated high metal uptake, reusability, and cost-effectiveness, making them attractive for wastewater treatment applications [17,18].

Moreover, algae biomass offers advantages over synthetic resins by effectively adsorbing metals even at low concentrations [19,20]. Furthermore, it can make up for the shortcomings of commercial resins, which lower sorption efficacy in wastewater with lower metal concentrations [21]. Dead cells may absorb more metals than living cells, according to the majority of research on the effectiveness of algal dry material biomass in metal removal [22].

Macroalgae have further potential due to their rich composition of bioactive compounds with antioxidant, antimicrobial, and other beneficial properties [23,24]. This versatility makes them suitable for innovative approaches such as nanobiotechnology, where their extracts can serve as reducing and stabilizing agents in the green synthesis of nanoparticles [25,26,27,28]. The bioactive metabolites present in abundance in algae extracts serve as stabilizing and reducing agents during the nanoparticle synthesis process [2,29,30]. Among these, silver nanoparticles (AgNPs) are particularly notable due to their high catalytic activity, stability, and optical properties, which enable applications in water treatment and pollutant degradation [31,32].

The synthesis of algal nanoparticles is a three-step process. Initially, an algal extract is prepared in either water or an organic solvent. Subsequently, molar solutions of ionic metallic compounds are generated. Finally, the algal solutions and molar solutions are incubated under controlled conditions [33]. Despite the potential of algae-based materials and nanotechnology, optimizing adsorption processes remains challenging due to the numerous factors influencing efficiency, such as pH, adsorbent dose, and temperature. Effective screening studies, such as one-factor-at-a-time (OFAT) and minimum-run resolution IV (MRR-IV) designs, can identify critical parameters and minimize experimental runs, reducing costs while maintaining reliability [34,35,36,37].

This study pioneers the comparative analysis of biosorption using *Enteromorpha intestinalis* and its extract-coated silver nanoparticles for ferrous ion removal. By applying OFAT and MRR-IV screening tools, this study investigates key factors, including pH, ferrous ion concentration, contact time, biosorbent dose, and temperature. This research aims to evaluate the efficacy of algal biomass and nanoparticles, hypothesizing that nanoparticle incorporation enhances adsorption efficiency under optimized conditions. This approach not only contributes to advancing sustainable water treatment technologies, but also underscores the role of green nanotechnology in addressing environmental challenges.

## 2. Experimental Procedure

### 2.1. Enteromorpha Intestinalis Water Extract (EiE)

*Enteromorpha intestinalis* (Linnaeus) Nees1820 was collected from the coastline of the Gulf of Suez in Egypt. The algae was identified by Dr. Fekry Ashour Mourad, a researcher at the National Institute of Oceanography and Fisheries (NIOF) in Egypt. All samples were identified in accordance with the previously established procedures and the methodology proposed by Aleem (1978) [35] and Silva (2002) [38]. These identifications were subsequently verified by the 2022 Algae Base website. Subsequently, the samples were rinsed with running water and distilled water to remove any residual sand, associated biota, and clinging debris. Following a period of air drying in a shaded environment, the *E. intestinalis* was subjected to an oven-drying process for a duration of three hours at a temperature of 60 °C (Memmert GmbH + Co. KG, Schwabach, Germany). Following the grinding of the samples in a Brown mill coffee grinder and their storage at room temperature in plastic bags, 10 g of algal powder was mixed with 100 mL of deionized water and heated for 30 min while stirring. Additional research was initiated using the supernatant.

### 2.2. Preparation of Enteromorpha intestinalis L. Extract (EiE) Silver Nanoparticles

To create *Enteromorpha intestinalis* L. AgNPs (*EiAgNPs*), 90 mL of a 1 mM silver nitrate (AgNO₃, Sigma Aldrich, St. Louis, MO, USA) solution was combined with 10 mL of pure *E. intesinalis* extract in a 250 mL conical flask. The mixture was mechanically stirred with a magnetic stirrer for 48 h to ensure the complete reduction of metal ions, and the experiment was conducted in a dark environment to minimize the photoactivation of silver nitrate. As soon as the reaction commenced, the formation of nanoparticles was first discernible by a change in hue from yellowish-brown to a concentrated dark brown [39], preceding the regular detection of the sample using a UV-vis spectrophotometer in a wavelength range between 300 nm and 700 nm [40]. Prior to undergoing further analysis, the synthesized silver nanoparticles were subjected to repeated centrifugation at 20,000× *g* for 30 min (5430R, Eppendorf, Hamburg, Germany), cleaned with sterile Milli-Q VR water, and vacuum-dried.

### 2.3. Characterization

Ultraviolet (UV) scan analysis of *EiAgNPs* was conducted using a double beam UV–visible spectrophotometer (Thermo Scientific Evolution TM300, Thermo Fisher Scientific, Waltham, MA, USA) in the wavelength range of 340 nm to 700 nm. Additionally, an optical evaluation of the reactivity of the silver nitrate solution with the algal extract was performed.

Fourier transform infrared (FTIR) spectroscopy was employed at a resolution of 4 cm^−1^ and a wavelength range of 4000–400 cm^−1^ to investigate the presence of active groups on the surface of *EiAgNPs*. For the residue of the aqueous extract of *E. intestinalis*, a quantity of 5 mg was combined with KBr (of spectroscopic grade) powder and compressed into a pellet of 1 mm thickness for the purpose of transmission measurement. A transmission electron microscope (TEM) was employed with an accelerating voltage of 50 kV using a JEOL JEM-2100 (JEOL Ltd., Tokyo, Japan) to ascertain the dimensions and shape of the silver nanoparticles. Upon deposition of a drop of the *EiAgNPs* solution onto a carbon-coated copper grid, the water evaporated. The sample was repeatedly cleaned with distilled water in order to eliminate any contaminants.

X-ray diffraction (XRD) analysis was conducted to confirm the crystalline nature and phase composition of *EiAgNPs*. This analysis was performed using a scanning electron microscope (SEM) model JSMIT100, manufactured by JOEL, Japan, which was equipped with a LINXEYE detector. The instrument was operated at an accelerating voltage of 30 kV and an emission current of 10 mA, with radiation from a Cu anode (k = 1.54184 Å). In order to ascertain the approximate size of the *EiAgNPs*, Scherrer’s equation [41] (Equation (1)) was employed:

(1) $$D=\frac{K\lambda }{\beta \cos\theta }$$

where D is the average particle size, K is a constant usually equal to 0.9, λ = 1.54056 Å is the wavelength of CuKα radiation, β is the full width at half-maximum (FWHM) intensity of the peak in radian, and θ is Bragg’s diffraction angle.

Gas chromatography–mass spectroscopy (GC-MS) analysis of *E. intestinalis* methanol extract was conducted using (Thermo Scientific TRACE 1310) attached with an ISQ LT single quadrupole. Acquisition parameters: the initial temperature of the oven was 40 °C/3 min, which ramped from 5 °C/min to 280 °C, wait 5 min; the temperature of the injector = 200 °C, source temperature = 300 °C, transfer temp = 250 °C, column (DB5-MS, 30 m; 0.25 mm ID (J&W Scientific, Folsom, CA, USA)). Scan = 50–500 Da, the ratio of splitting = 20:1, injection volume = 1.0 μL of *E. intestinalis* sample, helium gas used as the carrier with flow (1 mL per minute). Solvent delay = 5 min. A Malvern, Particle Sizing Systems, Inc. Santa Barbara, CA, USA, was employed for analyzing zeta potential.

### 2.4. Biosorption Study

#### 2.4.1. Biosorption Equilibrium

*EiAgNPs* and *EiE* solutions were centrifuged at 20,000 rpm for 30 min to obtain pellets and dried under vacuum. A stock solution of 2000 mg/L of Fe^2+^ of ferrous sulfate was prepared. The solution was diluted in order to obtain the requisite solutions, which had concentrations of 2000, 1000, 500, and 250 mg/L. The experiment was conducted in 250 mL flasks containing varying amounts of biosorbent (EiE and EiAgNPs) at concentrations of 5, 10, 20, and 30 mg (previously prepared and dried) in 100 mL ferrous solution at varying concentrations. The pH of the medium was adjusted to 2.0, 4.0, 5.0, and 7.0 using 0.1 N HCl and 0.1 N NaOH solutions. The flasks were agitated at 200 rpm at room temperature for varying time periods: 30, 60, 90, 120, 150, 180, 210, and 240 min. Samples were centrifuged (4000 rpm, 20 min), and the residual Fe^2+^ in the supernatant solutions was determined using an atomic absorption spectrophotometer. The removal rate was evaluated as follows:

Removal (%) = (C_o_ − C_e_)/C_o_ × 100 (2)

where C_o_ is the initial metal ion concentration (mg/L), C_e_ is the residual metal concentration in solution (ppm).

The impact of some factors that affect the biosorption of ferrous ions, like the initial solution pH, ferrous ion concentration, contact time, and biomass dose, were evaluated to determine whether changes in these variables during the adsorption process significantly affected the removal % of Fe^2+^.

#### 2.4.2. Adsorption Isotherms

Langmuir [42] and Freundlich [43] are two sorption isotherm models that were applied to study the extract’s Fe^2+^ adsorption capacity. These isotherms were used to evaluate the extract materials’ surface characteristics and affinity, as well as to compare their adsorptive capacities. Langmuir and Freundlich are presented by the following equations (Equations (3) and (4)):

(3) $$\frac{C_{e}}{q_{e}}=\frac{1}{K_{L}}+\frac{a_{L}}{K_{L}}C_{e}$$

(4) $$\log q_{e}=\log K_{F}+\frac{1}{n}\log C_{e}$$

where a_L_ and K_L_ are the Langmuir isotherm constants, K_F_ is the Freundlich constant, and n is the Freundlich exponent.

#### 2.4.3. Kinetics Studies

The experimental data were analyzed using both the non-linear pseudo-first-order and non-linear pseudo-second-order kinetic models in order to clarify the mechanism controlling the biosorption of ferrous cations on the En extract. This analysis was conducted for Fe^2+^ adsorption onto En extract at varying initial concentrations of the ferrous ions. The non-linear equations for the pseudo-first-order [44] and pseudo-second-order [45] kinetic models are presented in Equations (5) and (6), respectively:

(5) $$\log\;(q_{e}-q_{t})=\log q_{e}-\frac{k_{1}\cdot t}{2.303}$$

(6) $$\frac{t}{q_{t}}=\frac{1}{k_{2}\cdot q_{e}^{2}}+\frac{t}{q_{e}}$$

where q_e_ and q_t_ represent the amounts of dye adsorbed (mg g^−1^) at equilibrium and at time t (min), respectively; k_1_ is the rate constant for adsorption (min^−1^) according to the pseudo-first-order; and k_2_ is the pseudo-second-order rate constant (g·mg^−1^min^−1^).

### 2.5. Experimental Design Using MRR-IV

Screening effects with MRR-IV serve as a preliminary step for multivariate optimization, with the objective of identifying the key factors influencing the process [46]. The efficacy of minimum-run resolution IV in experimental design, particularly in accelerating the elimination of organic contaminants and metals, sets it apart from other screening techniques, including fractional and full factorial design, Taguchi design, and Plackett–Burman design. In the present study, five potential factors that may influence the bioremediation of polluted water were investigated (Table 1). These factors were pH, concentration of ferrous ions, dose of biosorbent, contact time, and temperature. Each factor was tested at two levels, high (+1) and low (−1), to assess its impact on Fe^2+^ removal. Following the guidelines for minimum-run resolution IV, 20 trials were conducted, consisting of 12 factorial points. Each run was conducted through two replicas. To ensure experimental randomization, each run was carried out independently in a separate block. Design-Expert^®^ Software, Version 10 (DX10) from Stat-Ease, Inc. (Minneapolis, MN, USA) was used to analyze the data.

## 3. Result and Discussion

### 3.1. Characterization of Enteromorpha intestinalis L. Extract (EiE) and Its Extract-Coated Silver Nanoparticles

**Figure 1:**
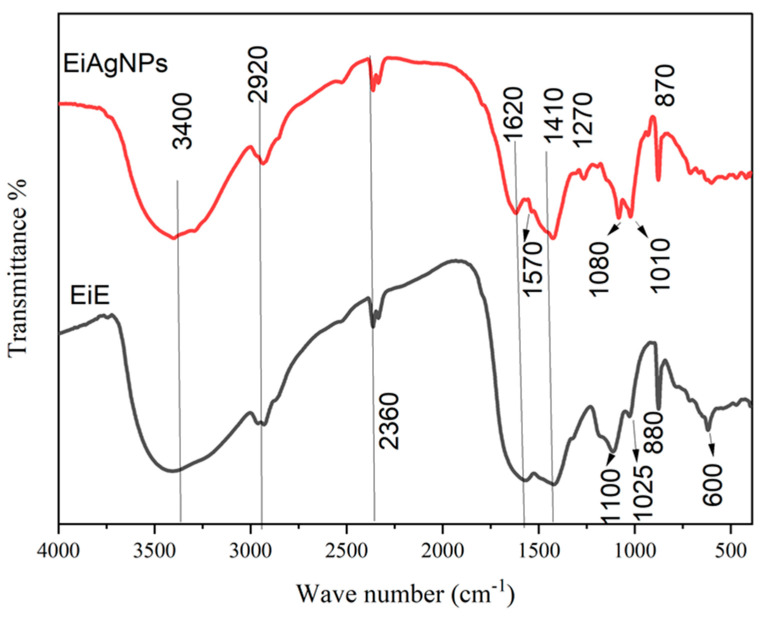
FTIR of *Enteromorpha intestinalis* L. aqueous extract (EIE) and its extract-coated silver nanoparticles. EiAgNPs.

**Figure 2:**
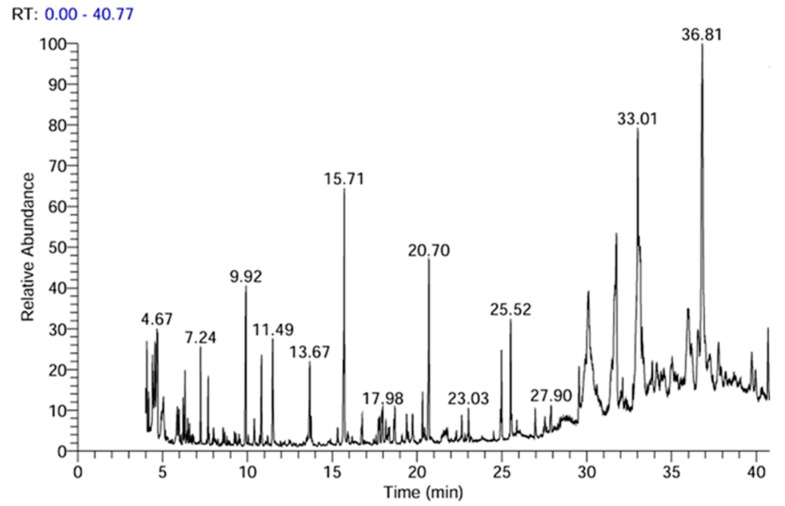
GC chromatogram of *Enteromorpha intestinalis* L. methanol extract. (EIE).

FTIR of *Enteromorpha intestinalis* extract and its prepared silver nanoparticles was conducted and is reported in Figure 1. There is a large degree of similarity between the *E. intestinalis* and *EiAgNPs* FTIR figures. The interaction between the algal extract component and the formed AgNPs is illustrated by the FTIR spectra, which exhibit nearly identical sharp absorption bands at 3400, 2920, and 2360 cm^−1^, exhibiting a slight degree of shifting. Moreover, the emergence of new peaks between 1070 and 880 cm^−1^ indicates the resonance of AgNPs. The FTIR spectra (Figure 1) and GC analysis (Figure 2) verified the function of bioactive substances found in the algal aqueous extract, which include primary and tertiary amines, amino acids, polysaccharides, and others. These substances serve as reducing, capping, and stabilizing agents for AgNPs [46]. The presence of an absorption band at 3400 to 3430 cm^−1^ of strong stretching–OH, as observed in the GC-MS analysis presented in Table 2, indicates the potential presence of alcohol or phenol molecules. [47]. The N=C=S stretching bond (isothiocyanate) and the CH₂ antisymmetric stretching of methyl groups in lipids are represented by the peak at 2970 to 2920 cm^−1^ [48]. The absorption bands at 1620 cm^−1^ show the stretching vibration of -C=O- of amide I, such as Oleamide, Trifluoroacetamide, and N-trimethylsilyloxymethyl- from Table 2, which is consistent with Demir [49] and is caused by the N-H bending vibration of secondary amines and the carbonyl unsaturated ketone amide (lipid, protein). The absorbance band at 1570 cm^−1^, which represents the stretching vibration of C=C, indicates the presence of lignin or the asymmetric stretching vibration of nitro compounds (N-O) [50]. The absorption band appeared at 1400 to 1410 cm^−1^, indicating the existence of carboxylic acid (O-H bending), such as 3-Methyl-2-furoic acid. Seaweed was recognized to contain a variety of carboxylic acids, particularly fatty acids such as palmitoleic acid and oleic acid nitrile [51]. The 1245 cm^−1^ absorbance band indicates the N-H stretching of primary and secondary amines, including ethylamine, Tritylamine, Mitomycin C, Benzyl-(4-chloro-benzyl)-amine, N-(1-Naphthyl) ethylenediamine, and Phenyl-ethanolamine triTMS (Table 2) [52]. Singh and ElSaied [52,53] claim that the absorbance band at 1070 cm^−1^ shows the stretching vibration of the C-N of aliphatic amines, as well as the stretching of the C-O of carbohydrates such as pectin and starch. Carbohydrate CH_2_OH groups are responsible for the 1100 cm^−1^ band [54]. The stretching of C=S in the absorption bands at 600 cm^−1^ (Chlorophyta) indicates the presence of sulfides in the examined algae. These results suggest that phenol, alcohol, lipids, proteins, fatty acids, and other phytochemicals were present in *E. intestinalis* and aided in the conversion of silver nitrate to AgNPs [55].

**Figure 3:**
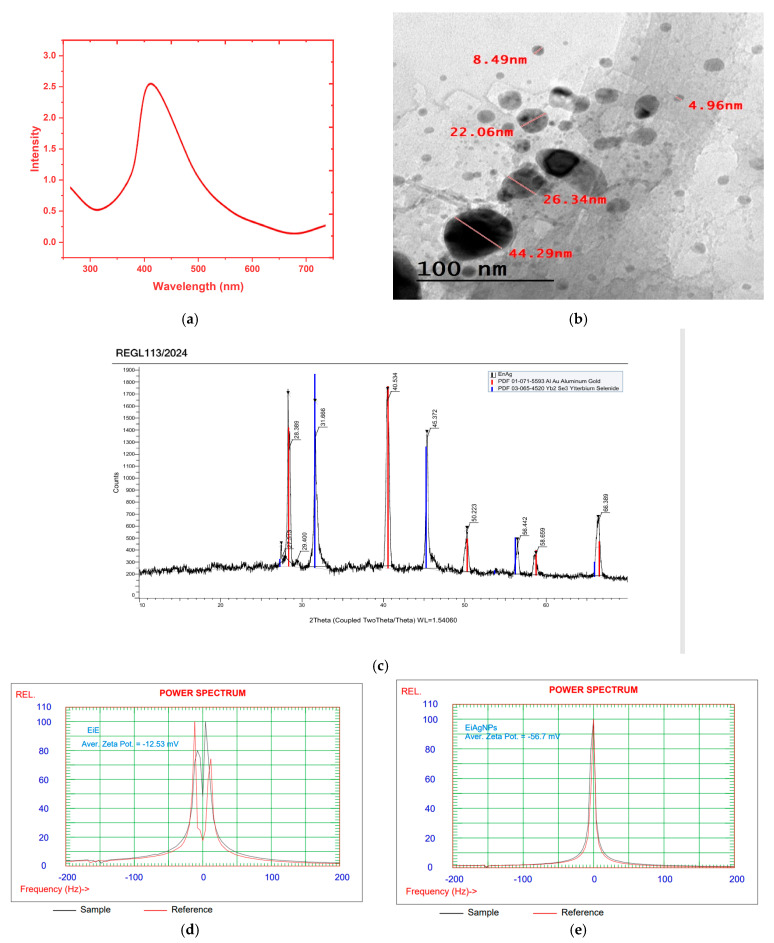
UV spectroscopy (**a**), TEM photographs (**b**), XRD spectrum (**c**), and zeta potential (**d**,**e**) of *Enteromorpha intestinalis* L. extract EIE and EiAgNPs.

To confirm the production of AgNPs, the Surface Plasmon Resonance (SPR) vibration was detected at 430 nm (Figure 3a). When the aqueous extract of E. intestinalis was added, the colorless silver nitrate solution turned a distinctive reddish-brown hue and conveniently excited SPR vibrations, a sign that AgNPs were forming. The absorption of AgNPs between 410 and 440 nm has also been found by many other researchers and has been attributed to the SPR of AgNPs [56].

The crystalline structure, size dispersion, shape, and surface condition of the synthesized AgNPS are among the physical and chemical characteristics that have been shown to influence their application [57]. AgNPs’ size and shape were determined by TEM measurements, which revealed that they had smooth spherical and semi-spherical morphologies and ranged in size from 4.69 nm to 44.29 nm (Figure 3b). These results support those of earlier studies [58,59], who synthesized AgNPs using *Enteromorpha intestinalis* and *Hormophysa triquetra* extracts, respectively. Transmission electron microscopy (TEM) revealed that the AgNPs exhibited a smooth, spherical, or semi-spherical morphology with a uniform distribution. The crystal structure and phase composition of AgNPs were determined by applying the XRD methods illustrated in Figure 3c. As evidenced by the XRD pattern, the sample is nanocrystalline, thereby indicating the formation of silver nanoparticles. The diffraction peak observed in the diffraction pattern (JCPDS card number 65-1290) is consistent with a particular diffraction peak in the 28.3–66.3° range at the angle of 2θ. It can be posited that the particles obtained are composed of silver. The narrowness of the diffraction peak indicates that the synthesized nanoparticles are crystalline, of a very small size, and exhibit weak amorphous characteristics. The main peaks of crystalline AgNPS are visible in the XRD patterns at 2θ values of 28.3°, 31.6°, 40.5°, 45.3°, 50.2°, 56.4°, 58.6°, and 66.3° (Figure 3c) corresponding to the crystal planes (025), (131), (400), (242), (237), (600), (520), and (440), respectively. Scherrer’s equation yielded an average crystalline size of 16.8 nm for biogenic Ag nanostructures, which is consistent with TEM findings. The presence of extra bioactive chemicals in the *E. intestinalis* extract may have contributed to the background noise generated by the biological approach of AgNPs (Figure 3c). According to Ferdous et al. (2024), the powder’s XRD pattern shows that post-annealing is not required to produce the desired crystalline phase [60].

### 3.2. Factors Affecting Biosorption

#### 3.2.1. Effect of pH

**Figure 4:**
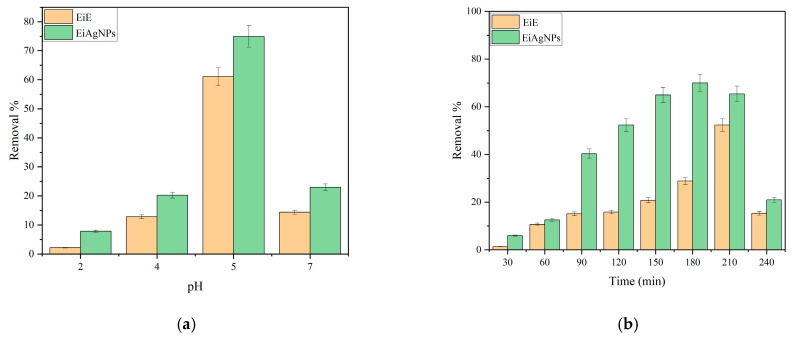

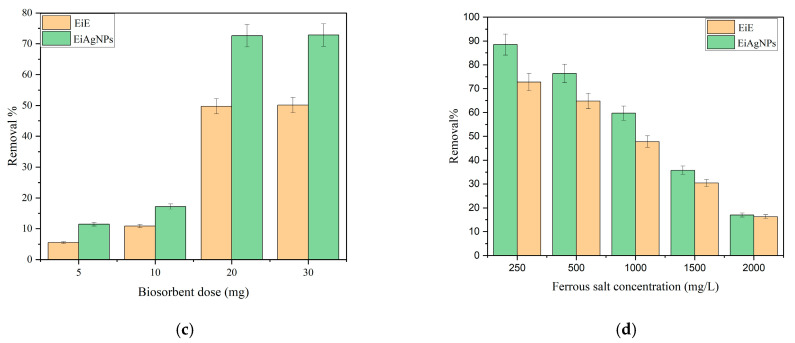
Effect of pH (**a**), effect of time (**b**), effect of dose (**c**), and effect of Fe^2+^ concentration (**d**) of EiE and EiAgNPs.

The experiment was conducted using 100 mL of ferrous solution and initial biosorbent concentrations of 1000 mg/L (previously prepared and dried). The experiment was carried out in 250 mL flasks. The medium was diluted with 0.1 N HCl and 0.1 N NaOH to achieve pH levels of 2, 4, 5, and 7. The flasks were agitated at room temperature at 200 rpm for a period of three hours. The atomic absorption spectrophotometer was employed to quantify the residual iron in the supernatant solutions following a 20-min centrifugation at 4000 rpm. The data obtained from the three trials were averaged to determine the mean values and standard errors. Figure 4 illustrates the impact of various parameters on the biosorption of ferrous ions. Figure 4a illustrates that the pH of the solution exerts an influence on the biosorption process, with the greatest degree of removal occurring at pH 5, which is a relatively acidic medium. This outcome aligns with the zeta potential assessment data, which indicate that the values for EiE and *EIAgNPs* are −12.53 and −56.7 mV, respectively (Figure 3d,e). The negative zeta potential suggests that the solid’s surface is rich in negatively charged groups, which will attract positively charged species (Fe^2+^) from the surrounding medium. This is important in applications such as water purification, biosorption, and catalysis.

#### 3.2.2. Effect of Contact Time

The practical application of any adsorbent material is contingent upon equilibrium time, which represents a crucial factor. The experiment was conducted in 250 mL flasks with 10 milligrams of biosorbent in a 100 mL ferrous solution to ascertain the influence of contact time on the biosorption process. The pH of the medium was adjusted to 5.0 using 0.1N HCl and 0.1N NaOH solutions, in accordance with the value determined in the preceding experiment. The flasks were agitated at room temperature at 200 rpm for a period of three hours. Samples were collected at various time points (30, 60, 90, 120, 150, 180, 210, and 240 min) and subsequently subjected to centrifugation. The residual Fe^2+^ in the supernatant solutions was determined. Figure 4b shows the effect of contact time on Fe^2+^ removal. Fe^2+^ removal increases gradually with time and the maximum removals are found to be 52% and 70% at 210 min and 180 min for EiE and *EiAgNPs*, respectively. It can be observed that equilibrium is reached within 210 and 180 min for the two biosorbents EiE and *EiAgNPs*, respectively, followed by a decrease in the removal percentage. The initial increase in the removal percentage up to the equilibrium time can be attributed to the progressive occupation of active sites on the adsorbent surface by the Fe^2+^ contaminant, which reaches a point close to equilibrium. Following the attainment of equilibrium, the observed decline in removal efficiency is presumably attributable to the saturation of active sites, the desorption of iron, or competitive adsorption.

#### 3.2.3. Effect of Biosorbent Dose

The efficacy of the adsorbent dose was assessed by varying the quantity of the adsorbent between 5 and 30 mg. One hundred milliliters of ferrous solution was combined with varying quantities of the adsorbent, and the experiments were conducted at room temperature with a pH of 5 and a contact time of three hours. As illustrated in Figure 4c, the data demonstrate that the removal rate of Fe^2+^ ions is enhanced with an elevated dose of the adsorbent for both materials. This increase in removal rate reaches its maximum value (49.8 and 72.6 for EiE and *EiAgNPs*, respectively) when the dose of the adsorbent is 20 mg. At higher doses, the number of active sites available for metal ion interaction increases, resulting in a higher removal percentage. Upon increasing the dose of biosorbent to 30 mg, no discernible increase was observed, with the removal percentage reaching 50.1 and 72.8 for EiE and *EiAgNPs*, respectively. This suggests that the biosorbent surface may have reached a saturation point.

#### 3.2.4. Effect of Initial Fe^2+^ Concentrations

The experiment was conducted using Fe^2+^ concentrations of 2000, 1500, 1000, 500, and 250 mg/L, with all other parameters held constant. These parameters included a 10 mg dose of biosorbent, a pH of 5, a contact duration of 3 h, and room temperature. The experiment was carried out in 250 mL flasks. Upon examination of ion removal at the initial concentration (Figure 4d), a discernible increase in removal is observed across both biosorbents under study. As evidenced by the calculations, when the initial ion concentration increased from 46 to 360 mg/L, the amount absorbed per gram of Fe^2+^ exhibited a notable increase, from 33 mg to 59 mg (for EiE) and from 41 to 62 mg (for EiAgNPs). Nevertheless, when the removed amount is recalculated and expressed as a percentage of the initial ion concentration, a different trend becomes evident. Specifically, the removal of Fe^2+^ decreased from 72.8% to 16.3% and from 88.5% to 17% for EiE and EiAgNPs, respectively, when the initial ion concentration was increased from 46 to 360 mg/L. Fluctuations in the observed results can be attributed to two factors: the fixed number of adsorption sites on the adsorbent and the decreased ion concentration in the solution at lower starting concentrations [61].

### 3.3. Screening Study by MRR-IV

In order to optimize the factors affecting the removal of ferrous cations, a screening process based on the MRR-IV method was implemented. In the screening study, five parameters were investigated, namely the initial Fe^2^⁺ concentration, biosorbent dose, solution pH, contact time, and temperature. Each parameter was tested at two levels: low (−1) and high (+1) (see Table 1). This configuration yielded 12 preliminary experiments, with each experiment replicated twice to yield a total of 24 runs. The overall number of experiments conducted for the two adsorbents was 48. The dependent variable was the percentage of ferrous ion removal.

**Figure 5:**
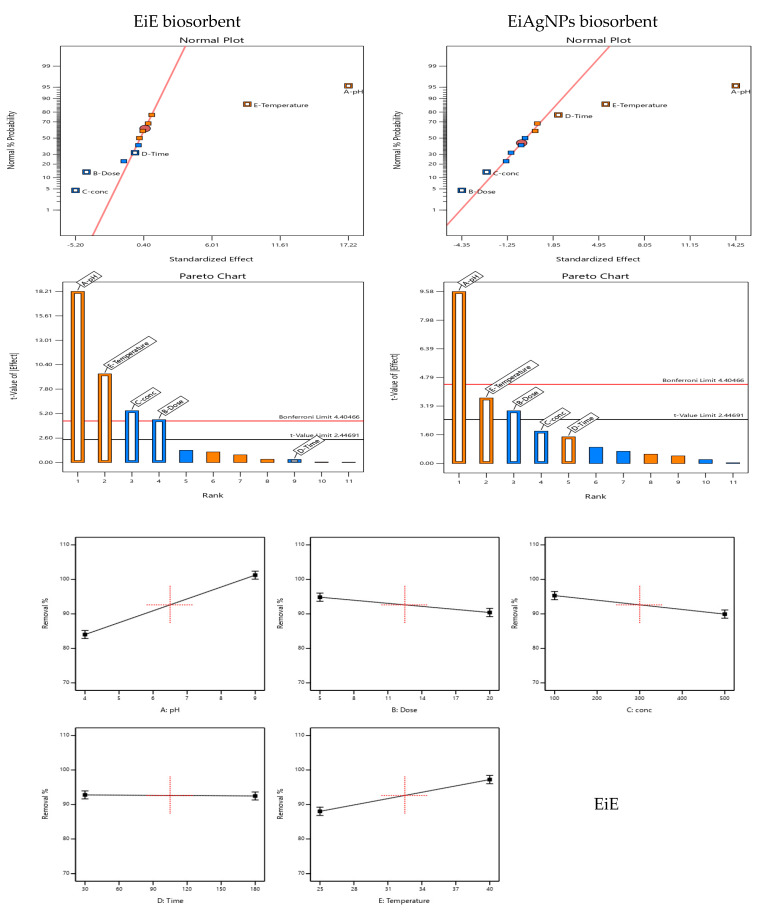

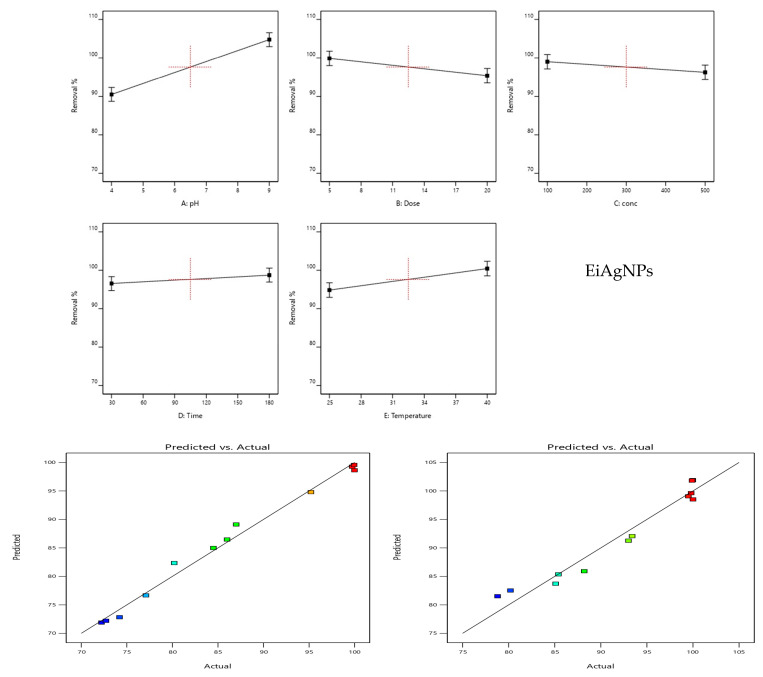
MRR-IV design plots of Fe^2+^ removal (%), including normal plots, Pareto charts, interactions plots, and predicted vs. actual plots for both EiE and EiAgNPs biosorbent., Color points by value of removal % 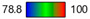.

The results of the analysis of variance (ANOVA) employed to evaluate the efficacy of the MRR-IV design are presented in Table 3 (a) and (b). The model F-values of 106.50 and 23.56 indicate that the model is statistically significant for both biosorbents, EiE and *EiAgNPs*, respectively. The model terms are deemed significant if the *p*-value is less than 0.0500, and conversely, not significant if the *p*-value is greater than 0.1000. In the case of the EiE biosorbent, only pH, dose, and Fe^2^⁺ concentration were identified as significant model terms. In contrast, the significant model terms for the *EiAgNPs* biosorbent are pH, dose, and temperature. Furthermore, the ANOVA demonstrated that the adjusted R^2^ of 0.98 and 0.90 and the predicted R^2^ of 0.95 and 0.71 are in reasonable agreement for both biosorbents, indicating a difference of less than 0.2. The Adeq Precision ratio of 25.3 and 10.2 for the EiE and *EiAgNPs* biosorbents, respectively, indicates an appropriate signal, thereby enabling the utilization of the model to explore the design space. Adeq Precision assesses the signal-to-noise ratio, with a ratio greater than 4 being preferable [62]. The precision and dependability of the experiments are confirmed by the CV% values of 1.8 for EiE and 2.86 for EiAgNPs, which show little variation between the actual and predicted values (Table 3 and Table 4, and Figure 5) [63]. Then, Fe^2+^ removal using both EiE and EiAgNPs can be described by Equation (7) and Equation (8), respectively.

(7) Removal%=58.23+3.44A−0.2956B−0.0134C−0.0021D+0.6137E

(8) Removal%=71.27+2.85A−0.297B−0.0069C+0.0147D+0.3729E

**Table 3:** (a): ANOVA for the selected factorial model for EiE biosorbent. (b): ANOVA for the selected factorial model for EiAgNPs biosorbent.

Source	Sum of Squares	df	Mean Square	F-Value	*p*-Value	
(a)						
Model	1269.74	5	253.95	106.50	<0.0001	significant
A-pH	790.60	1	790.60	331.56	<0.0001	
B-Dose	49.15	1	49.15	20.61	0.0039	
C-conc	72.12	1	72.12	30.24	0.0015	
D-Time	0.2546	1	0.2546	0.1068	0.7549	
E-Temperature	211.87	1	211.87	88.85	<0.0001	
Residual	14.31	6	2.38			
Cor Total	1284.05	11				
(b)						
Model	693.79	5	138.76	23.52	0.0007	significant
A-pH	541.31	1	541.31	91.75	<0.0001	
B-Dose	50.54	1	50.54	8.57	0.0264	
C-conc	18.96	1	18.96	3.21	0.1232	
D-Time	12.94	1	12.94	2.19	0.1892	
E-Temperature	78.23	1	78.23	13.26	0.0108	
Residual	35.40	6	5.90			
Cor Total	729.19	11				

(a): Std. Dev 2.63; R^2^ 0.95; Adjusted R^2^ 0.90; Predicted R^2^ 0.71 Adeq Precision 10.2, and C.V.% 2.86. (b) Std. Dev 1.54; R^2^ 0.99; Adjusted R^2^ 0.98; Predicted R^2^ 0.95; Adeq Precision 25.34, and C.V.% 1.8.

**Table 4:** Design matrix and the results of the fractional factorial design.

Run	pH	Dose (mg)	Fe Conc. (mg/L)	Time (min)	Temp. (°C)	Removal % En Extract		Removal % Ag Extract	
						Actual	Predicted	Actual	Predicted
1	2	20	100	30	40	80.2	82.35	88.2	85.91
2	7	5	500	30	40	99.98	98.63	99.98	101.90
3	7	5	100	30	25	95.2	94.79	99.5	99.06
4	2	5	500	30	25	72.711	72.20	78.8	81.52
5	2	20	500	180	40	77.09	76.67	85.4	85.36
6	2	20	100	180	25	74.2	72.83	80.2	82.52
7	7	20	100	30	40	99.94	99.57	99.8	99.62
8	7	20	500	30	25	84.5	84.99	93	91.27
9	7	5	500	180	25	87	89.11	100	98.51
10	2	5	500	180	25	72.22	71.90	85.1	83.72
11	7	20	100	180	40	99.73	99.26	99.9	101.82
12	2	5	100	180	40	86	86.47	93.4	92.07

Figure 5 illustrates the normal plot of standardized effects and the Pareto chart, which are commonly employed to identify the primary effects of independent variables, interactions, and between-variable relationships, as well as predicted versus actual plots. The diagnostic plots served to further confirm the significance of the selected factors. In the normal probability plots for the five factors (Figure 5), factors with linear lines near the effects are statistically insignificant, whereas factors whose effects deviate notably from the linear lines are statistically significant [64]. The significant factors identified for EiE biosorbents were pH, temperature, and Fe^2+^ concentration. For EiAgNPs, the significant factors were pH, temperature, and dose. Furthermore, the Pareto chart of standardized effects (Figure 5) indicates that the significant factors exert a greater influence than the other factors. This suggests that for the EiE biosorbent, the pH, Fe^2+^ concentration, and temperature are the most influential factors in the proposed 2FI design model, while for the EiAgNPs, the pH, temperature, and dose are the most influential factors [65]. Consequently, these three factors (for each biosorbent) were selected as optimized conditions, while the other less significant parameters were excluded.

### 3.4. Adsorption Equilibrium Isotherms

**Figure 6:**
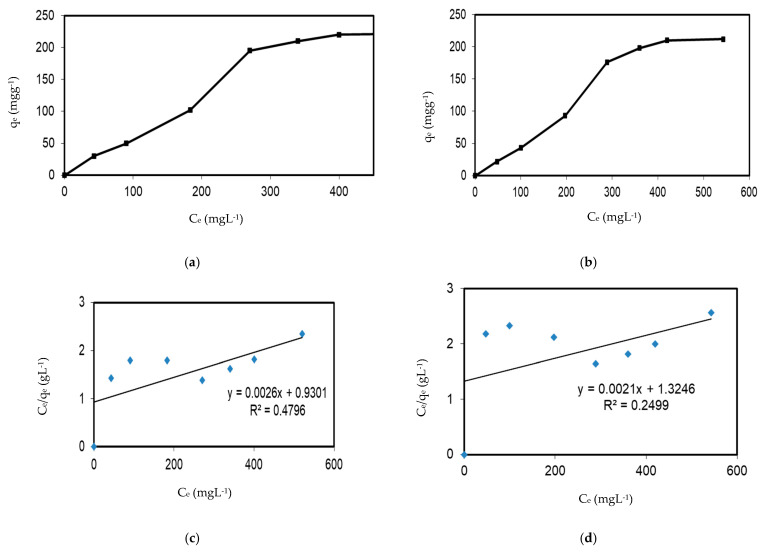

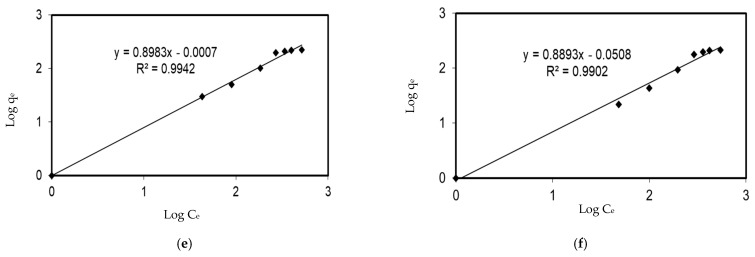
Adsorption isotherms (**a**,**b**), Langmuir isotherms (**c**,**d**), and Freundlich isotherms (**e**,**f**) for EiE and EiAgNPs, respectively.

The analysis of adsorption equilibrium isotherms, which describe the relationship between the concentration of adsorbate in the liquid phase and the solid phase, provides valuable insights into the properties of the adsorbent. When the equilibrium adsorption capacity (q_e_) and equilibrium concentration (C_e_) were plotted against the experimental data points of ferrous cations’ adsorption onto biosorbents, characteristic S-shaped curves were observed (for both biosorbents) according to Giles’ classification system [66], as shown in Figure 6a,b. The aforementioned curves classify the isotherms as type-S, which typically indicates cooperative adsorption. This phenomenon is characterized by the adsorption of the initial molecules increasing the likelihood of further adsorption at neighboring sites [67]. The shape of the isotherm indicates that the initial affinity between the Fe^2+^ and the biosorbent is relatively low. However, as more Fe^2+^ molecules bind, the affinity increases due to interactions between the Fe^2+^ molecules. S-shaped isotherms are frequently associated with multilayer adsorption or systems where adsorbate molecules interact strongly with one another, thereby enhancing the overall adsorption process as coverage increases.

The adsorption equilibrium data were modeled using the linear, Langmuir, and Freundlich isotherms. These models assist in determining whether the adsorption surface is heterogeneous or homogeneous and in evaluating the extent of interactions between adsorbed species. Accordingly, the experimental data on Fe^2^⁺ removal by the synthetic biosorbents were analyzed using the Langmuir and Freundlich isotherms, as detailed in Equations (3) and (4). The Langmuir isotherm model describes the adsorption process as occurring in a monolayer, thereby illustrating the distribution of metals between the solid and liquid phases. A summary of the results obtained from both isotherms is presented in Table 5. The R^2^ values for the Freundlich isotherm were 99% for both biosorbents, while the correlation coefficients for the Langmuir isotherm were very small, at 0.48 and 0.25 for EiE and *EiAgNPs*, respectively. These results indicate that the Freundlich isotherm is applicable to this process. The n parameter for the Freundlich isotherm is greater than one, with values of 1.11 and 1.12 for EiE and *EiAgNPs*, respectively. This indicates that the biosorption process is favorable.

### 3.5. Kinetic Study

**Figure 7:**
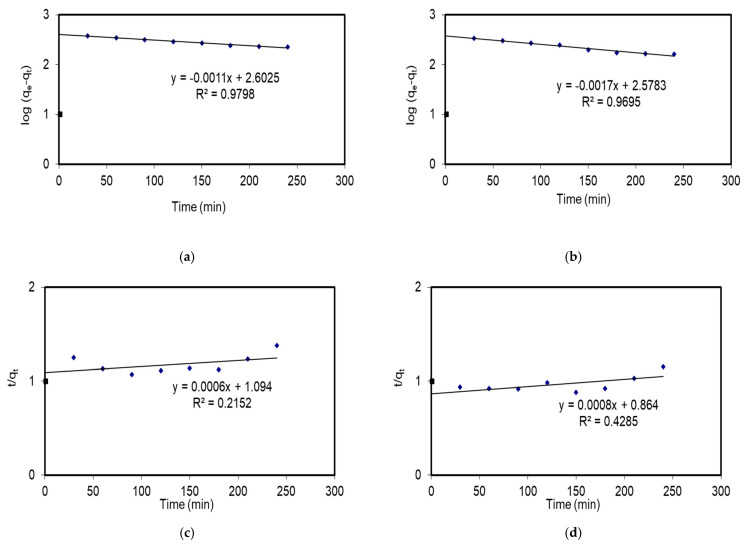
Adsorption kinetics (**a**,**b**), pseudo-first order, and (**c**,**d**) pseudo-second order for EiE and EiAgNPs, respectively.

The data parameters for kinetics are presented in Table 6, and the kinetic models for both pseudo-first-order and pseudo-second-order adsorption processes are shown in Figure 7.

As illustrated in Figure 7a,b, both biosorbents demonstrate high regression coefficients (R^2^), with values exceeding 0.9 for the pseudo-first-order kinetic model, indicating a robust fit to the model. This suggests that the number of available (unoccupied) adsorption sites on the adsorbent is directly correlated with the rate of adsorption.

This model postulates that the rate at which the adsorbate (the substance undergoing adsorption) binds to the adsorbent (the material performing the adsorption) is predominantly influenced by the concentration of unoccupied sites [68]. On the contrary, Equation (6) was used to obtain the pseudo-second-order rate constant values (k2), which are shown in Table 6 and Figure 7c,d.

The regression coefficients (R^2^) for the pseudo-second-order model were found to be 0.22 and 0.43 for EiE and *EiAgNPs*, respectively, indicating that this model is not applicable in this context. In light of the aforementioned evidence, it can be definitively concluded that the biosorption of Fe^2^⁺ follows the pseudo-first-order model.

## 4. Conclusions

This study successfully demonstrated the synthesis and application of cost-effective and environmentally friendly biosorbents derived from the aqueous extract of *Enteromorpha intestinalis* and its extract-coated silver nanoparticles (*EiAgNPs*) for the removal of Fe^2^⁺ ions from aqueous solutions. The comprehensive characterization of these biosorbents revealed their nanoscale size, high degree of crystallinity, and the presence of various bioactive components, including fatty acids, organic acids, alcohols, and terpenes, which were confirmed through FTIR and GC-MS analyses.

The adsorption studies revealed that a number of variables exert a significant influence on the biosorption process. The application of a minimum-run resolution IV (MRR-IV) design proved an effective method for identifying the most critical factors affecting Fe^2^⁺ removal. These were found to be pH, biosorbent dosage, and Fe^2^⁺ concentration for the aqueous extract (EiE), and pH, biosorbent dosage, and temperature for the silver nanoparticles (*EiAgNPs*). The optimal conditions for maximum Fe^2^⁺ removal were determined, with the biosorption process following the Freundlich isothermal model and pseudo-first-order kinetics.

It is noteworthy that the biosorbents exhibited remarkable adsorption capacities, achieving maximum capacities of 386 mg g⁻^1^ for EiE and 375 mg g⁻^1^ for *EiAgNPs*. These findings highlight the potential of *Enteromorpha intestinalis* and its extract-coated silver nanoparticles as sustainable and efficient materials for the removal of metals from contaminated water. Furthermore, the study underscores the significance of optimizing biosorption conditions to enhance removal efficiency, which can make a substantial contribution to environmental remediation efforts.

Future research should concentrate on the scaling up of the biosorption process for industrial applications, the exploration of the regeneration and reuse of the biosorbents, and the investigation of the removal of other metals and contaminants using these biosorbents. Moreover, a comprehensive assessment of the environmental impact and safety of biosynthesized nanoparticles is essential to guarantee their sustainable integration into water treatment technologies.

## Figures and Tables

**Table 1:** Levels of variables and experimental range.

Variables	Unit	Code	Levels	
			Low (−1)	High (+1)
pH	-	A	2	7
Extract Dose	Mg	B	5	20
Fe concentration	mg/L	C	100	500
Contact time	Min.	D	30	180
Temperature	°C	E	25	40

**Table 2:** GC analysis of *Enteromorpha intestinalis* L. methanol extract. (EIE).

RT	Compound	Common Name	Molecular Formula	Peak Area %	M. wt	Chemical Group
5.87	2-Phenyl-1-[1-(1-Pyrrolidinyl) Cyclohexyl]	Dextromethorphan	C_18_H_25_NO	0.90	271	
4.42	N-(Hydroxymethyl)trifluoroacetamide, TMS	Trifluoroacetamide, N-trimethylsilyloxymethyl-	C_6_H_12_F_3_NO_2_Si	1.44	215	Amide
29.55	9-Octadecenamide	Oleamide	C_18_H_35_NO	1.12	281	
4.55	Benzamine-Chloro-N-Ethyl-N-Phenyl	Benzyl-(4-chloro-benzyl)-amine	C_14_H_14_ClN	2.19	231	Amine
4.68	1,1′-Dimethyl-4,4′-dipyridinium	N-(1-Naphthyl)ethylenediamine	C_12_H_14_N_2_	3.70	186	
23.03	4-Trimethysilyloxyphenneth YL-N,N-Bis(trimethylsilyl)Amine	Phenylethanolamine triTMS	C_17_H_35_NOSi_3_	0.66	353	
34.12	6,6,7-Trimethyl-9-oxo-3-oxabicyclo(3.3.1)nonane 2,4-dinitrophenylhydrazone	Mitomycin C, ethylamine	C_17_H_22_N_4_O_5_	1.33	362	
36.81	[1,1′-Biphenyl]-2-Amine, N-(4-methylphenyl)	Tritylamine	C_19_H_17_N	10.54	259	
6.33	Bistrimethylsilyllactic acid	Trimethylsilyl (2S)-2-[(trimethylsilyl)oxy]propanoate	C_9_H_22_O_3_Si_2_	1.12	234	Organic acid
9.91	Urea, N,N’-bis(trimethylsilyl)-	Bis(trimethylsilyl)harnstoff	C_7_H_20_N_2_OS_i2_	3.57	402	
10.83	3,7-Dioxa-2,8-disilanonane, 2,2,8,8-tetramethyl-5-	Trimethylsilyl ether of glycerol	C_12_H_32_O_3_Si_3_	1.86	308	
16.77	Uridine, 2′,3′,5′-tris-O-(trimethylsilyl)-	Uridine, 2′,3′,5′-tris-O-TMS	C_18_H_36_N_2_O_6_S_i3_	0.69	460	
11.49	Furan-2-carboxylic acid, 3-methyl-, trimethylsilyl ester	3-Methyl-2-furoic acid, TMS	C_9_H_14_O_3_Si	5.52	198	carboxylic acid
13.67	trans-4-Trimethylsilyloxy-cyclohexyl(tr imethylsilyl)carboxylate	2-Ethyl-3-ketovalerate, bis(trimethylsilyl)	C_13_H_28_O_3_Si_2_	2.02	288	Ester
15.71	(t-Butyldimethylsilyl)[3-methyl-3-(4-me thyl-pent-3-enyl)-oxiran-2-yl]-methano ne	Palmitoleic Acid	C_16_H_30_O_2_Si	5.34	282	Fatty acid
25.52	Oleanitrile	Oleic acid nitrile	C_18_H_33_N	2.09	263	
24.98	12-Methoxy-2-Trimethylsilylodyloxy-19-Nor-5á-Podocarpa-1,3,8,11,13-pentene	Isotretinoin	C_20_H_28_O_2_Si	1.89	328	Conjugated acid
27.90	Deoxyherqueinone	Cubebin	C_20_H_20_O_6_	0.68	356	alcohol
38.68	Stigmadta-5,24(28)-Dien-3-OL, Acetate, (3á,24E)	Suberosol	C_31_H_50_O_2_	0.68	454	terpenoid

**Table 5:** Langmuir and Freundlich parameters.

	Langmuir Parameters						Freundlich Parameters		
Adsorbent	K_L_ (Lg^−1^)	a_L_ (Lmg^−1^)	q_max_ (mg·g^−1^)	q_ref_ (mg·g^−1^)	C_ref_ mg·L^−1^	R^2^	K_F_ (Lg^−1^)	n	R^2^
EiE	1.08	0.003	386	400	220	0.48	0.98	1.11	0.99
EiAgNPs	0.75	0.002	375	370	200	0.25	0.89	1.12	0.99

**Table 6:** Kinetic parameters for pseudo-first and pseudo-second orders.

Biosorbent	Pseudo-First Order		Pseudo-Second Order	
	K_1_ (min^−1^)	R^2^	K_2_ (g·mg^−1^min^−1^)	R^2^
EiE	2.53 × 10^−3^	0.98	5.7 × 10^−6^	0.22
EiAgNPs	3.9 × 10^−3^	0.97	8.5 × 10^−6^	0.43

## Data Availability

Data are contained within the article.
